# Saint Thomas's Hospital

**Published:** 1829-01-01

**Authors:** 


### St. Thomas's Hospital.

#### I. Encysted Tumours on the Scalp.

The constant occurrence of encysted tumours in various parts of the body, especially the head, renders the best mode of treating them a matter of no little importance to the surgeon. When the cyst can be removed without wound to its walls or extravasation of its contents, the operation is undoubtedly best and most effectually performed. This, however, in certain situations, is difficult, sometimes impossible, as in those encysted tumours which occur about the eye-lids of children, in which the parietes are exceedingly thin. In these cases, or at least when a puncture has been made, the readiest method is to cut through the centre of the cyst, and successively dissect out either portion, by means of the forceps and knife. When suppuration has occurred, it frequently happens that opening the tumour, and dressing it with lint, induce ulceration or sloughing of the cyst, and thereby effect a cure. In the following instances a different and curious result was the consequence.

*Case* 1. A young woman was admitted into St. Thomas's Hospital, with a hemispherical tumour on the upper part of the forehead, as large as a pullet's egg. She said that it had existed from her infancy, had been hard till a few weeks before her admission, but had lately become softer. Fluctuation being present, Mr. Green made a transverse incision in the tumour, giving vent to a quantity of puriform matter. The cavity being lined with a proper membrane, it was sprinkled with red precipitate, and filled with dry lint, in order to excite inflammation, and cause the opposing surfaces of the cyst to adhere. The result was, however, very different indeed from what had been contemplated and intended by the surgeon.

At the end of a month, the interior of the cavity was covered with a cuticle, continuous with that upon the scalp, and presenting the same general characters and properties. The appearance was remarkable, and somewhat disagreeable, as the sides, being elastic, were partially erect.—*Gaz.*

A similar case occurred to Mr. Green in the person of a child, which was born with a tumour, filled with fluid, opposite the ischiatic notch. It was opened, the cyst took on the character of skin, forming a permanent unsecreting cavity, seated in one of the nates. Mr. Green was afraid to have recourse to caustic, on account of the vicinity of the deepest part of the cyst to the pelvic viscera, whilst the proximity of the cavity to the bone of the cranium deterred him from operating in the former case.

We almost believe that the cyst on the forehead would be found to be above the occipito-frontalis, and therefore amenable to the surgical knife. We have seen a very old and rather large-encysted tumour of the scalp, treated with perfect success by dissecting out the membrane. This was converted in places into bone, and several lines in thickness. Tumours containing fluid occasionally form in various parts of the body, painful on pressure, often very numerous, and dubious in their nature. On puncturing them and evacuating their contents by pressure, or the suction of a cupping-glass, these are found to consist of a yellow-coloured fluid, glairy in consistence, and presenting flakes or masses of solid albumen or lymph, floating in and greatly thickening its consistence. Whether these should be considered as suppurating bursæ or mere encysted tumours, we will not pretend to decide. Sometimes merely puncturing the tumour, extracting its contents by means of a cupping-glass, and afterwards applying a blister to the surface, succeeds in removing the swelling. We have had no opportunity of dissecting them or witnessing their removal in the living body.

### ST. THOMAS'S HOSPITAL.

[Reporter, Mr. BURY.]

I. DISLOCATION OF THE THIGH-BONE INTO THE ISCHIATIC NOTCH.—Ben. Whittenburg, æt. 40, a very stout and rather tall countryman, was admitted, Nov. 4th, with a dislocation of the right hip, of twenty-two weeks' duration. The injury was occasioned, during the performance of his labour, by a large piece of timber striking against his shoulders, coming also in contact with the outside of the lower part of the thigh, whilst he was falling forwards to the ground from the force of the blow upon the upper part of his body. His right arm was also fractured at the same time. In this, there is much overlapping of the fractured extremities, with shortening of the limb.

The following are the appearances and state of the lower extremity of the affected side. A considerable eminence is produced posteriorly below the crista ilii, by the displaced head of femur, which is situated higher than ordinarily in the dislocation backwards. It admits of an unusual degree of motion, can be distinctly felt resting a little above the ischiatic notch upon the glutæus medius muscle, and, when rotated, its movements are distinguishable beneath, or anteriorly to, some of the fibres of the glutæus maximus. The distance from the anterior superior spinous process of the ilium to the patella is found, by measurement, to be nearly two inches less on the right side than on the left. The right knee and foot are inverted, and the toes are approximated to the ball of the other foot. Flexion can be performed upwards to some extent, but scarcely at all backwards, independent of the movements of the pelvis. The different motions of the leg, made either by the surgeons or the patient himself, produce but little pain. This has latterly been unimportant, but was severe for some time after the injury was first inflicted.

No attempts have ever been made to replace the bone; but, from the mobility of its head, and other circumstances of the case, it was considered advisable, even at this late period, to endeavour to reduce the dislocation.

Nov. 7th. Warm bath and venesection in the arm having been employed, the patient was brought into the operating theatre, well covered with blankets, &c. and was placed on a table upon his back. The usual bandage and padded leather belt, to which the strings of the pullies are attached, were fixed just above the knee. The pelvis was secured by a girt, placed between the scrotum and thigh in the ordinary manner. Extension was made almost, if not quite, directly downwards, *i.e.* in the line of the body, and was gradually increased in force during forty-five minutes, the time employed in the operation. Rotation outwards and inwards was, at the same time, performed, both from knee and ankle. An antimonial solution was given at intervals, which shortly produced nausea and tendency to vomit. More blood was also taken from both arms pretty freely. An alteration in the situation of the head of the femur, and in the length of the limb likewise, slowly and gradually took place a short time after these means were used; and about forty minutes having elapsed since the extension was commenced, the head of the bone had moved immediately posterior to the acetabulum. The upper extremity of the femur was now elevated by a towel placed under it, and drawn upwards and forwards; the extension and rotation were continued, and, the head of the bone being firmly pressed upwards and forwards by Mr. Travers, its return into the socket was effected, and plainly heard. The patient himself was conscious of its replacement.

A curious circumstance was observable for nearly half an hour after the reduction; namely, shortening of the thigh, with inversion of the leg, as previously to it. After the patient had been in bed, however, a short time, both patellæ were brought together, and the right was half an inch above the left; the toes of right foot turned inwards.

8th. Patient had little or no sleep during the night; says he feels weak from the loss of blood; is in no pain, except in the loins, which has existed since he left the operating table, and has no fever; there is some puffiness behind the trochanter major; he can rotate the foot outwards and inwards, which he has not been able to do before since the accident, but he does not move the knee at the same time. The limb certainly does not bear the appearance of dislocation, although the patellæ are uneven, and the inversion is the same. But, on measuring the space, from spinous process to patella, on each side, that on the right is found shorter than that on the opposite by 1¾ inches. This proves that the femur is now dislocated; and, on inquiry, it is learned from the patient, that he moved in the night on his left side, from which cause, it may be supposed that the change of position in the bone took place, though quite unconsciously to the patient. The head of the bone can be distinguished completely in the ischiatic notch.

11th. Pain in the loins has been severe, but does not at present exist—pain across the bottom of abdomen during the last day or two, but no where near to the hip—tumefaction diminished—same appearance of the limb. Has had head-ache, and a quickened pulse, which are relieved by a calomel purge.

13th. The displaced bone was again tried to be reduced, but without success. In addition to the means practised on the former occasion, Mr. Travers made considerable efforts in rotating the femur outwards and inwards, whilst the extension was intermitted, by taking hold of the thigh, below its middle, with both hands. A considerable time having expired in these ineffectual attempts. some change had obviously occurred, for the limb was perfectly moveable, and, in fact, had lost all features of dislocation; and, on further examination, a crepitus was discovered at the cervix femoris. Fracture having unfortunately ensued, the patient was placed upon Mr. Amesbury's bed, with the extremity on an inclined plane, and the pelvis fixed by the strap.

It appears probable that the head and neck of the bone had undergone absorption to some extent in their newly-formed situation, and thus the facility of fracture may be accounted for, since no more than ordinary force was made use of.

14th. The hip-joint has been very painful since yesterday, but he obtained some hours' sleep in the night—head in pain—is thirsty, and feels uncomfortable—bowels not open.

15th. In less pain, and feels much better—had some sleep—head easy—pulse firm—bowels relieved by medicine. There is no tenderness of the hip. When left to the unrestrained action of its muscles, the thigh becomes shortened to nearly two inches, but by gentle extension with the hand at the ancle a difference of an inch and a half is produced, the whole limb being then only half an inch shorter than natural. The head is situated below the ischiatic notch. It seems doubtful whether the whole sphere of the head of the bone is detached from the cervix, as far as can be judged by manual examination. Mr. Travers and Mr. Amesbury, the circumstances of the case being new to them, and having no data from experience of similar cases on which to act, agreed upon bringing the upper end of the bone as near to the acetabulum as they could, and upon keeping it there by means of Mr. A's apparatus. With this view, extension of the extremity is persevered in upon the inclined plane, the foot being secured at the foot-board by bandage to maintain due extension of the leg. Extended in this manner, the thigh is abbreviated about ¾ of an inch. Mr. Travers conjectures that the fracture of the neck happened during the performance of rotation outwards. Some restorative process, it is reasonable to expect, will be set up by Nature, and by placing the fractured surface near to acetabulum, it is also reasonable to suppose that the best restoration, whether in the form of joint, or any other likely to be useful for the purposes of motion, will be effected in this the situation of the natural joint. Some months must necessarily elapse before the result can be known.

II. DISLOCATION OF THE RADIUS FORWARDS AND UPWARDS, AND FRACTURE OF THE ULNA, NEAR TO OLECRANON.—Timothy Carthy, æt. 40, whilst engaged at work, fell into a cellar from a height of some feet, Oct. 1st. and came upon his right shoulder, but whether or not his arm was bent under him, he does not recollect. The elbow-joint was a good deal swollen, and in great pain. The limb was placed horizontally upon a pillow, and leeches and cold lotion were applied.

Oct. 4th. Great swelling of the joint—pain felt particularly severe on pressure near the outer condyle, and on the posterior surface of fore-arm, not far from olecranon. The patient has no power of raising the limb, nor can any the slightest motion be performed without intense pain. The head of radius is resting upon the outer condyle anteriorly; the olecranon is drawn upwards, a sharp projection being formed at the back of the elbow, with a depression a little below, produced by the separation of the broken extremities of the ulna. Mr. Green exerted gentle extension from the hand for a few minutes, trying to replace the radius. He pointed out the singularity of the injury, and observed, that the indication now was, to reduce the inflammation of the part by ordinary means, before again attempting reduction. Patient complains only of his elbow; pulse rather quick; bowels opened by house medicine. Rep. hirud. et lotio.

Oct. 10th. Tumefaction subsiding, but patient complains greatly of pain—pulse natural—a thick paste-board splint is applied. Lotion continued.

15th. Swelling and inflammation are much reduced. Pretty forcible extension, supination being at the same time employed, was again made from the carpus, or rather extremities of radius and ulna, but the head of radius was not apparently moved. The fracture of ulna prevented the use of any excessive degree of force.

20th. Parts kept at rest, with paste-board splint and lotion applied.

27th. Hitherto the joint has been easy. Reduction was once more attempted, by placing the fore-arm at an angle with the arm, making pressure with the knee against the lower part of the humerus, and by employing extension and supination from above the wrist, but without effect. The mode adopted before was also tried, but with no better result. Paste-board splints were used above and below the joint, which was kept at rest on a pillow. No inflammation ensued.

Nov. 13th. There is no pain in the limb, or joint, except on motion. The power of motion is at present very limited. The ulna, at its seat of fracture, we should suppose, must be nearly consolidated. Wooden splints are kept on the fore-arm.

